# Clinical characteristics and outcomes of pregnancies at-risk of hemolytic disease of the fetus and newborn in Sweden, Finland, and Denmark: a population-based register study

**DOI:** 10.1016/j.xagr.2025.100544

**Published:** 2025-07-08

**Authors:** Kelvin H.M. Kwok, Mika Gissler, Mette Ø. Thunbo, Elizabeth C. Hsia, May Lee Tjoa, Shengxin Liu, Malin Almgren, Vedran Stefanovic, Lars H. Pedersen, Agneta Wikman

**Affiliations:** 1 Schain Research AB, Stockholm, Sweden (Kwok, Liu, and Almgren); 2 Department of Data and Analytics, Finnish Institute for Health and Welfare, Helsinki, Finland (Gissler); 3 Department of Molecular Medicine and Surgery, Karolinska Institutet, Stockholm, Sweden (Gissler); 4 Department of Clinical Medicine, Aarhus University, Aarhus, Denmark (Thunbo and Pedersen); 5 Department of Clinical Pharmacology, Aarhus University Hospital, Aarhus, Denmark (Thunbo); 6 Johnson & Johnson, Spring House, PA (Hsia); 7 Johnson & Johnson, Cambridge, MA (Tjoa); 8 Department of Medical Epidemiology and Biostatistics, Karolinska Institutet, Stockholm, Sweden (Liu); 9 Fetomaternal Medical Centre, Department of Obstetrics and Gynecology, Helsinki University Hospital and University of Helsinki, Helsinki, Finland (Stefanovic); 10 Department of Obstetrics and Gynecology, Aarhus University Hospital, Aarhus, Denmark (Pedersen); 11 Clinical Immunology and Transfusion Medicine, Karolinska University Hospital, Stockholm, Sweden (Wikman); 12 Department of Hematology and Regenerative Medicine, MedH, Karolinska Institutet, Stockholm, Sweden (Wikman)

**Keywords:** Alloimmunization, hemolytic disease of the fetus and newborn, intrauterine transfusion, neonatal outcomes, population-based study, pregnancy outcomes

## Abstract

**Background:**

Red blood cell (RBC) alloimmunization is an immune response where the maternal immune system produces antibodies against fetal RBCs, which can lead to hemolytic disease of the fetus and newborn (HDFN). Despite the significant clinical burden of HDFN, there are few large international cohorts that focus on perinatal care and outcomes of at-risk pregnancies.

**Objective:**

To describe the maternal characteristics and outcomes of pregnancies affected by RBC alloimmunization, as well as the characteristics and outcomes of neonates from such pregnancies.

**Study Design:**

Utilizing data from nationwide health registers, this population-based cohort study identified all singleton pregnancies in individuals who had ≥1 pregnancy monitored or treated for potential alloimmunization, or ≥1 child with a postnatal diagnosis of HDFN-related conditions, between January 1, 2000, and December 31, 2021, in Sweden and Finland, and between January 1, 1997, and December 31, 2018, in Denmark. Among the identified pregnancies, those with a diagnosis of maternal care for alloimmunization or fetal hydrops, or neonates with a postnatal diagnosis of HDFN-related conditions, were categorized as HDFN pregnancies. The remaining pregnancies—sibling pregnancies that may have been at risk of alloimmunization but did not receive any alloimmunization- or HDFN-related diagnosis—were categorized as non-HDFN pregnancies.

**Results:**

This study included 14,732 singleton pregnancies in Sweden, 5863 in Finland, and 11,964 in Denmark. Among these pregnancies, 7391 (50%) in Sweden, 2885 (49%) in Finland, and 6150 (51%) in Denmark were categorized as HDFN pregnancies. Maternal complications and stillbirth rates were comparable between HDFN and non-HDFN pregnancies. Caesarean deliveries were more frequent in HDFN pregnancies. A total of 14,519 neonates in Sweden, 5827 in Finland, and 11,803 in Denmark were born to all pregnancies identified. Of these, 7289 (50%), 2849 (49%), and 6076 (51%) had HDFN. Among the neonates with HDFN, 27% in Sweden, 38% in Finland, and 12% in Denmark received HDFN-related treatment, including intrauterine transfusion (IUT; data unavailable for Finland), neonatal transfusion, and phototherapy. Compared to non-HDFN neonates, those in the IUT and neonatal transfusion groups had lower gestational age, birth weight and length, and higher rates of neonatal unit admission, and were more frequently diagnosed postnatally with growth disturbances and disorders of the nervous system.

**Conclusion:**

This is a comprehensive overview of perinatal characteristics and outcomes of pregnancies at risk of HDFN in Sweden, Finland, and Denmark. Our findings highlight the significant unmet need in perinatal care among neonates with HDFN, particularly those treated with IUT or neonatal transfusion. Further research is warranted to improve the management of severe HDFN pregnancies.

**AJOG Global Reports at a Glance**

**Why was this study conducted?**

To examine the maternal and neonatal outcomes of pregnancies at risks of hemolytic disease of the fetus and newborn (HDFN) across three Nordic countries.

**Key findings**

Pregnancies at risk of HDFN showed higher rates of caesarean deliveries and adverse neonatal outcomes, including lower birth weight and higher neonatal unit admissions, particularly for neonates requiring intrauterine or postnatal transfusions.

**What does this add to what is known?**

This study, one of the largest international cohorts on pregnancies at risk of HDFN, underscores the substantial unmet need in managing severe cases of HDFN, advocating for enhanced perinatal care to improve neonatal outcomes.

## Introduction

Red blood cell (RBC) alloimmunization is an immune response whereby the maternal immune system produces antibodies against fetal RBCs, which can be triggered by previous pregnancies, blood transfusions, or fetomaternal hemorrhage during the current pregnancy.^1^^,^^2^ RBC alloimmunization is an uncommon pregnancy event with a prevalence of roughly 1%, and it may lead to fetal RBC hemolysis and result in hemolytic disease of the fetus and newborn (HDFN).^2^^,^^3^

In its most severe form, HDFN may manifest as progressive fetal anemia and fetal hydrops. Severe HDFN can necessitate invasive interventions such as intrauterine transfusions (IUTs), which are aimed to correct the fetal anemia by providing compatible RBCs to the fetus.^4^ In contrast to the rare severe cases that require IUTs, there exists a broader spectrum of HDFN cases that may range in severity from mild to moderate and still require neonatal care. These neonates, although generally affected to a lesser extent, often present with symptoms such as anemia and jaundice which, if left untreated, can escalate to severe hyperbilirubinemia and kernicterus. Treatment for these neonates includes phototherapy and RBC transfusion, and less typically, exchange transfusions and intravenous immunoglobulin therapy (IVIg). These treatments are essential to prevent long-term complications, such as hearing loss, cerebral palsy, and neurodevelopmental disabilities, which may be associated with untreated or inadequately managed HDFN.^5^^,^^6^

Despite the significant efforts to monitor pregnancies at risk of HDFN and to reduce morbidity and mortality in neonates with HDFN, there remains a paucity of knowledge in the perinatal characteristics and outcomes of at-risk pregnancies.^7^ Using population-based register data from three Nordic countries, this study aimed to comprehensively describe the maternal clinical characteristics and outcomes of pregnancies at risk of HDFN, as well as birth characteristics and health outcomes of neonates born to these pregnancies.

## Material and methods

### Data sources

Data for this study was sourced from national population-based health registers in Sweden, Finland, and Denmark (Supplemental Information). In Sweden, linked data was obtained from the Medical Birth Register, Patient Register, Swedish Neonatal Quality Register, Cause of Death Register, and GravImm (www.gravimm.se), a clinical database containing medical records of RBC-alloimmunized pregnancies. In Finland, linked data was obtained from the Medical Birth Register, Care Register for Health Care, Social Insurance Institution, and Cause of Death Register. In Denmark, linked data was obtained from the Medical Birth Register, National Patient Register, National Health Services Prescription Database, and Cause of Death Register. For Sweden and Finland, data was available between January 1, 2000, and December 31, 2021; for Denmark, data was available between January 1, 1997, and December 31, 2018.

### Study population

We included all singleton pregnancies that occurred during the study period in individuals who fulfilled at least one of the following criteria: (1) had ≥1 pregnancy being monitored or treated for potential alloimmunization (ICD-10: O36.0–O36.2); and (2) had given birth to ≥1 neonate who received postnatal diagnosis of HDFN-related conditions (ICD-10: P55.0, P55.8, P55.9, P56.0, and P57.0) (Supplemental Table 1 and Supplemental Figure 1). Pregnancies were excluded if no recorded delivery date was available or the maternal age at delivery was below 15 years. Pregnancies ending before 22 weeks of gestation (28 weeks of gestation prior to July 1, 2008, in Sweden and April 1, 2004, in Denmark) were not registered at the national Medical Birth Registers of the countries examined, and were therefore excluded from this study.

All included pregnancies were followed up until the delivery date, date of emigration, or death of the pregnant individual, whichever came earlier. For pregnancies that ended with a live birth, neonates were further followed up from birth to the end of the study period, date of emigration, or death, whichever came earlier.

### Exposures

Pregnancies with a diagnosis record of maternal care for alloimmunization or fetal hydrops, or those resulting in a neonate with a postnatal diagnosis of HDFN-related conditions, were categorized as HDFN pregnancies. The remaining pregnancies, ie, sibling pregnancies which were at risk of alloimmunization but lacked any alloimmunization- or HDFN-related diagnoses, were categorized as non-HDFN pregnancies.

Neonates born from HDFN pregnancies were referred to as HDFN neonates hereafter, and were further stratified based on the type of perinatal treatment received (Supplemental Table 2), including (1) IUT, (2) neonatal transfusion (RBC and/or exchange transfusion), and (3) neonatal phototherapy. If a HDFN neonate received more than one type of treatment, they were classified based on the most intensive intervention, in the following order of intensivity: IUT>neonatal transfusion>phototherapy. HDFN neonates who did not have any record of the above treatments were assigned to the “unknown” treatment group.

### Outcomes

Key maternal clinical characteristics (including maternal age at delivery, parity, body mass index, maternal complications), intrauterine growth restriction, delivery methods, and pregnancy outcomes were described. In addition, clinical characteristics at birth and health outcomes of live births (including admission to neonatal unit, neonatal and long-term diagnoses, and mortality) were analyzed. Details of all examined outcomes and the corresponding ICD-10 are listed in Supplemental Table 3.

### Statistical analysis

Descriptive statistics were used to summarize maternal characteristics, pregnancy outcomes, and neonatal outcomes, presenting mean and standard deviation, or median and interquartile range, for continuous variables, and frequencies and percentages for categorical variables. Data management and data analyses were performed with R (version 4.2.3) and SAS (version 9.4) statistical software.

## Results

### Study population

A total of 15,282 pregnancies in Sweden, 6009 in Finland, and 12,275 in Denmark were identified. Among these, singleton pregnancies constituted 96% of all pregnancies in Sweden (*n*=14,732), 98% in Finland (*n*=5863), and 97% in Denmark (*n*=11,964) (Table 1). Distribution of perinatal diagnoses in the study population was largely comparable across all countries. Prenatal diagnoses of maternal care for alloimmunization or fetal hydrops were reported in 40% of singleton pregnancies in Sweden, 37% in Finland, and 45% in Denmark. Postnatal HDFN-related diagnoses were reported in 21% of neonates in Sweden, 24% in Finland, and 15% in Denmark. IUT was performed to 1.0% of pregnancies in Sweden and 1.2% in Denmark; no data was available in Finland.

**Table 1:** Summary of all pregnancies included in the study population

	Sweden	Finland	Denmark
**Pregnancies identified**	***n* (% of total pregnancies)**		
Total	15,282	6009	12,275
Singleton	14,732 (96)	5863 (98)	11,964 (97)
**Singletons where mother was treated or monitored for alloimmunization**	***n* (% of singleton)**		
Maternal care for rhesus isoimmunization	3659 (25)	1372 (23)	2709 (23)
Maternal care for other isoimmunization	2559 (17)	729 (12)	2897 (24)
Maternal care for fetal hydrops	274 (1.9)	216 (3.7)	414 (3.5)
Any of the above prenatal diagnoses of maternal care	5936 (40)	2185 (37)	5386 (45)
**Singletons where fetus received treatment**			
Intrauterine transfusion (IUT)	150 (1.0)	NA^a^	139 (1.2)
**Singletons where neonates received postnatal HDFN-related diagnosis**			
Rh isoimmunization of newborn	2051 (14)	1123 (19)	1232 (10)
Other hemolytic disease of newborn	581 (3.9)	196 (3.3)	401 (3.4)
Hemolytic disease of newborn, unspecified	493 (3.4)	113 (1.9)	238 (2.0)
Fetal hydrops due to isoimmunization	19 (0.1)	8 (0.1)	≤5 (≤0.04)
Kernicterus due to isoimmunization	25 (0.2)	9 (0.2)	8 (0.1)
Any of the above neonatal diagnoses	3029 (21)	1393 (24)	1818 (15)

Due to privacy regulations, values less than or equal to 5 are presented as “≤5.” In this table, some pregnancies were included in more than one category. Therefore, the sum of the number of cases per section and the corresponding percentages may appear more than the section’s total.

a Data for IUT not available in Finland.

### Maternal characteristics and pregnancy outcomes

Detailed maternal characteristics and pregnancy outcomes are summarized in Table 2. A higher proportion of HDFN pregnancies were multiparous (≥2) in Sweden (75% vs 54% for non-HDFN), Finland (80% vs 61% for non-HDFN), and Denmark (73% vs 50% for non-HDFN). Delivery by caesarean section (total) was more common in HDFN pregnancies, as observed in 25% of pregnancies in Sweden (vs 20% for non-HDFN), 30% in Finland (vs 21% for non-HDFN), and 31% in Denmark (vs 22% for non-HDFN). Maternal complications were largely comparable between HDFN and non-HDFN pregnancies, except for a higher incidence in gestational diabetes and placental previa in the former group. Stillbirth rate was consistently low (≤1.5%) in both HDFN and non-HDFN pregnancies across all countries.

**Table 2:** Maternal characteristics and pregnancy outcomes of singleton pregnancies included in the study population

	Sweden		Finland		Denmark	
	HDFN	Non-HDFN	HDFN	Non-HDFN	HDFN	Non-HDFN
**Singleton pregnancies, *n***	7391	7341	2885	2978	6150	5814
**Maternal age at delivery, y**						
Mean (SD)	31.8 (5.1)	29.6 (5.3)	31.9 (5.4)	29.0 (5.5)	31.3 (5.0)	29.1 (4.9)
**Parity (after delivery), *n* (%)**						
1	1818 (25)	3368 (46)	565 (20)	1161 (39)	1645 (27)	2847 (49)
2	2996 (41)	2198 (30)	1009 (35)	796 (27)	2511 (41)	1760 (30)
3	1620 (22)	1063 (15)	672 (23)	427 (14)	1332 (22)	752 (13)
4	572 (7.7)	377 (5.1)	435 (15)	340 (11)	434 (7.1)	276 (4.7)
≥5	385 (5.2)	335 (4.6)	203 (7.0)	251 (8.4)	194 (3.2)	112 (1.9)
Missing	0	0	≤5 (≤0.2)	≤5 (≤0.2)	34 (0.6)	67 (1.2)
**Body mass index, *n* (%)**						
Underweight (<18.5)	156 (2.1)	183 (2.5)	71 (2.5)	87 (2.9)	182 (3.0)	209 (3.6)
Normal weight (18.5–24.9)	3606 (49)	3790 (52)	1272 (44)	1353 (45)	2408 (39)	2240 (39)
Overweight (25.0–29.9)	1772 (24)	1737 (24)	518 (18)	518 (17)	945 (15)	794 (14)
Obese (≥30.0)	1069 (14)	986 (13)	423 (15)	307 (10)	632 (10)	516 (8.9)
Missing	788 (11)	645 (8.8)	601 (21)	713 (24)	1980 (32)	2055 (35)
**Maternal complications, *n* (%)**						
Gestational hypertension	99 (1.3)	100 (1.4)	115 (4.0)	118 (4.0)	110 (1.8)	95 (1.6)
Gestational diabetes	153 (2.1)	126 (1.7)	362 (13)	319 (11)	192 (3.1)	134 (2.3)
Proteinuria during pregnancy	7 (0.1)	7 (0.1)	7 (0.2)	8 (0)	24 (0.4)	13 (0.2)
Kidney disease associated with pregnancy	280 (3.8)	223 (3.0)	0 (0)	0 (0)	0 (0)	0 (0)
Preeclampsia	174 (2.4)	266 (3.6)	62 (2.1)	85 (2.9)	198 (3.2)	183 (3.1)
Eclampsia	≤5 (≤0.1)	≤5 (≤0.1)	0 (0)	≤5 (≤0.2)	7 (0.1)	3 (0.1)
Placental abruption	49 (0.7)	40 (0.5)	14 (0.5)	11 (0.4)	49 (0.8)	45 (0.8)
Placenta previa	40 (0.5)	29 (0.4)	45 (2.1)	39 (1.3)	74 (1.2)	43 (0.7)
PPROM	201 (2.7)	144 (2.0)	92 (3.2)	87 (2.9)	329 (5.5)	360 (6.2)
Obstetric embolism	≤5 (≤0.1)	≤5 (≤0.1)	0 (0)	0 (0)	≤5 (≤0.1)	≤5 (≤0.1)
**Prenatal diagnosis, *n* (%)**						
IUGR	159 (2.2)	148 (2.0)	126 (4.4)	107 (3.6)	501 (8.1)	202 (3.5)
**Delivery methods, *n* (%)**						
Vaginal	5504 (75)	5896 (80)	2025 (70)	2350 (79)	4226 (69)	4513 (78)
Caesarean						
Elective	898 (12)	577 (7.9)	419 (15)	261 (8.8)	827 (13)	419 (7.2)
Emergency	857 (12)	741 (10)	438 (15)	365 (12)	729 (12)	618 (11)
Missing caesarean delivery type	127 (1.7)	117 (1.6)	0 (0)	0 (0)	368 (6.0)	264 (4.5)
**Pregnancy outcomes, *n* (%)**						
Livebirth	7289 (99)	7230 (98)	2849 (99)	2978 (100)	6076 (99)	5720 (99)
Stillbirth	102 (1.4)	111 (1.5)	36 (1.0)	0 (0)	72 (1.2)	84 (1.4)
**Year of delivery, *n* (%)**						
1997–1999	NA^a^	NA	NA	NA	744 (12)	737 (13)
2000–2004	1227 (17)	1448 (20)	600 (21)	765 (26)	1367 (22)	1487 (25)
2005–2010	1877 (25)	2302 (31)	866 (30)	1140 (38)	1812 (30)	1948 (34)
2011–2015	2070 (28)	1892 (26)	831 (29)	683 (23)	1412 (23)	1199 (21)
2016–2020^b^	1835 (25)	1535 (21)	501 (17)	355 (12)	815 (13)	443 (7.6)
2021	382 (5.2)	164 (2.2)	87 (2.9)	35 (1.2)	NA	NA

Due to privacy regulations, values less than or equal to 5 are presented as “≤5.”

*HDFN*, hemolytic disease of fetus and newborn; *IUGR*, intrauterine growth restriction; *PPROM*, preterm premature rupture of membranes; *SD*, standard deviation.

a Data not available (NA)

b For Denmark, this category of year of delivery only covers pregnancies from 2016 to 2018.

### Perinatal treatment pattern in HDFN neonates

Among neonates born to HDFN pregnancies, 27% in Sweden, 38% in Finland, and 12% in Denmark received treatment for HDFN (including IUT [no data available in Finland], neonatal transfusion, or phototherapy) (Table 3, Figure). Across all countries, majority of neonates in the IUT (79%–95%) and neonatal transfusion groups (60%–68%) were associated with both maternal care for alloimmunization or fetal hydrops, and postnatal diagnosis of HDFN-related conditions (Supplemental Table 4). In contrast, among neonates in the unknown treatment group, 72% in Sweden, 70% in Finland, and 76% in Denmark were associated only with a prenatal diagnosis of maternal care for alloimmunization or fetal hydrops.

**Table 3:** Characteristics at birth of liveborn neonates included in the study population, by treatment groups

	Sweden					Finland					Denmark				
	IUT	Transfusion	Phototherapy	Unknown	Non-HDFN	IUT	Transfusion	Phototherapy	Unknown	Non-HDFN	IUT	Transfusion	Phototherapy	Unknown	Non-HDFN
**Total liveborn, *n***	149	517	1284	5339	7230	NA^a^	193	900	1756	2978	134	169	441	5332	5727
% of HDFN	2.0	7.1	17.6	73.2	-	NA	6.8	31.6	61.6	-	2.2	2.8	7.3	87.8	-
**Sex**															
Female	77 (53)	223 (43)	579 (45)	2628 (49)	3577 (49)	NA	72 (37)	391 (43)	823 (47)	1395 (47)	69 (51)	88 (52)	189 (43)	2513 (47)	2793 (49)
**Gestational age (wk)**															
Mean (SD)	36 (2)	36 (3)	38 (2)	39 (2)	38 (2)	NA	36 (3)	38 (2)	39 (2)	40 (2)	35 (3)	36 (3)	38 (3)	39 (2)	40 (2)
≤32	6 (4.1)	63 (12)	38 (3.0)	92 (1.7)	109 (1.5)	NA	25 (13)	24 (2.7)	53 (3.0)	33 (1.1)	17 (13)	23 (14)	22 (5)	126 (2)	82 (1)
33–34	12 (8.1)	56 (11)	53 (4.1)	88 (1.7)	87 (1.2)	NA	27 (14)	37 (4.1)	60 (3.4)	29 (1.0)	34 (25)	22 (13)	25 (6)	118 (2)	70 (1)
35–36	81 (54)	130 (25)	137 (11)	315 (5.9)	272 (3.8)	NA	38 (20)	153 (17)	143 (8.1)	105 (3.5)	49 (37)	28 (17)	50 (11)	269 (5)	145 (3)
≥37	50 (34)	268 (52)	1055 (82)	4841 (91)	6758 (93)	NA	103 (53)	646 (72)	1495 (85)	2803 (94)	34 (25)	96 (57)	344 (78)	4819 (90)	5430 (95)
**Apgar score**															
**1 min**															
Mean (SD)	8.4 (1.6)	7.7 (2.4)	8.6 (1.3)	8.7 (1.3)	8.6 (1.4)	NA	7.4 (2.4)	8.5 (1.3)	8.3 (1.7)	8.5 (1.3)	NA	NA	NA	NA	NA
7–10	130 (87)	398 (77)	1194 (93)	5032 (94)	6752 (93)	NA	142 (74)	837 (93)	1564 (89)	2791 (94)	NA	NA	NA	NA	NA
**5 min**															
Mean (SD)	9.3 (1.0)	8.8 (2.0)	9.7 (0.9)	9.7 (1.0)	9.6 (1.1)	NA	8.1 (1.6)	8.9 (1.0)	8.8 (1.2)	9.0 (0.9)	9.6 (1.2)	9.1 (1.9)	9.8 (0.6)	9.8 (0.9)	9.8 (0.9)
7–10	141 (95)	443 (86)	1262 (98)	5205 (98)	7039 (97)	NA	157 (81)	862 (96)	1634 (93)	2881 (97)	125 (93)	153 (91)	433 (98)	5177 (97)	5616 (98)
**Birth weight (g)**															
Mean (SD)	2907 (464)	2908 (752)	3345 (626)	3448 (578)	3381 (618)	NA	2934 (761)	3306 (629)	3407 (678)	3541 (587)	2590 (665)	2780 (759)	3096 (716)	3378 (616)	3495 (613)
<2500	18 (12)	112 (22)	119 (9.3)	263 (4.9)	317 (4.4)	NA	42 (22)	82 (9)	155 (9)	120 (4)	57 (43)	44 (26)	81 (19)	348 (6.3)	283 (4.9)
≥2500	130 (87)	399 (77)	1164 (91)	5052 (95)	6899 (95)	NA	151 (78)	817 (91)	1596 (91)	2856 (96)	77 (57)	122 (72)	356 (81)	4918 (92)	5399 (94)
**Birth length (cm)**															
Mean (SD)	48 (3)	48 (4)	50 (3)	50 (3)	50 (3)	NA	48 (4)	49 (3)	50 (3)	50 (3)	45 (12)	42 (17)	48 (10)	50 (8)	51 (6)
**SGA**	97 (65)	231 (45)	220 (17)	486 (9.1)	531 (7.3)	NA	67 (35)	179 (20)	185 (11)	155 (5.2)	≤5 (≤3.7)	≤5 (≤3.0)	≤5 (≤1.1)	≤5 (≤0.1)	≤5 (≤0.1)
**IUGR-associated SGA**	≤5 (≤3.4)	≤5 (≤1.0)	17 (1.3)	59 (1.1)	90 (1.2)	NA	≤5 (≤2.6)	13 (1.4)	28 (1.6)	25 (0.8)	≤5 (≤3.7)	≤5 (≤3.0)	≤5 (≤1.1)	≤5 (≤0.1)	0

Due to privacy regulations, values less than or equal to 5 are presented as “≤5.” The “% of HDFN” values are presented as “-” for non-HDFN groups.

*HDFN*, hemolytic disease of fetus and newborn; *IUGR*, intrauterine growth restriction; *IUT*, intrauterine transfusion; *SD*, standard deviation; *SGA*, small for gestational age.

a Data not available (NA).

**Figure:**
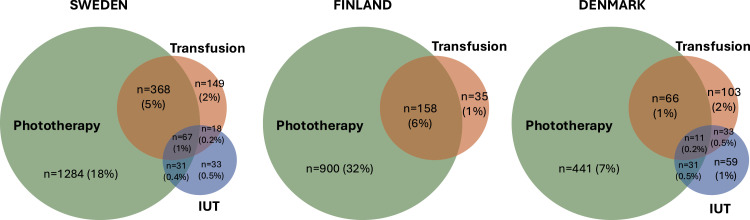
Treatment patterns in liveborn neonates with HDFN in Sweden, Finland, and Denmark The figure shows the number of neonates in each mode of treatment, and the respective percentages among all HDFN neonates (Sweden, *n*=7289; Finland, *n*=2849; Denmark, *n*=6076). *HDFN*, hemolytic disease of fetus and newborn; *IUT*, intrauterine transfusion.

Kwok. Clinical characteristics and outcomes of pregnancies at-risk of hemolytic disease of the fetus and newborn in Sweden, Finland, and Denmark. AJOG Glob Rep 2025.

### Characteristics at birth

The characteristics at birth of all neonates are detailed in Table 3. In all countries, the proportion of preterm birth (<37 weeks gestational age) increased with intensity of perinatal treatment (ie, IUT>neonatal transfusion>phototherapy>unknown treatment). The proportion of very preterm birth (≤32 weeks gestational age) was highest in the neonatal transfusion group (12%–14%).

Mean Apgar scores were generally lower in the IUT and neonatal transfusion groups, and the lowest 5-minute Apgar scores were observed in the neonatal transfusion group in all countries. Birth weights and lengths showed similar patterns, where lower mean values were observed in the IUT and neonatal transfusion groups.

### Neonatal and long-term outcomes

Admission rates to neonatal units were higher in neonates with HDFN compared to non-HDFN neonates across all countries, with the highest found in the neonatal transfusion group (89%–94%) (Table 4). Congenital anemia was more often diagnosed in the IUT (5.4%–11%) and neonatal transfusion groups (3.1%–8.5%). Neonatal jaundice was more prevalent in the phototherapy group (27%–61%) within each country. Kernicterus was uncommon (≤1.2%) across all groups and countries. Neonatal infections were more prevalent in the IUT (5.4%–11%) and neonatal transfusion groups (11%–13%) in both Sweden and Denmark, whereas very few cases were reported in Finland (*n*≤5 in all groups). Neonatal mortality was reported in ≤2.6%, 0.8%, and 0.5% the transfusion, unknown treatment, and non-HDFN groups in Finland, respectively, and 3.9%, 1.0%, and 0.4% in Sweden, respectively. No neonatal death was reported across all groups in Denmark. Cumulative incidence of the examined long-term outcomes was generally low (Table 5). There were no clear differences observed between HDFN and non-HDFN children.

**Table 4:** Neonatal outcomes of liveborn children included in the study population, by treatment groups

	Sweden					Finland					Denmark				
	IUT	Transfusion	Phototherapy	Unknown	Non-HDFN	IUT	Transfusion	Phototherapy	Unknown	Non-HDFN	IUT	Transfusion	Phototherapy	Unknown	Non-HDFN
Total liveborn, *n*	149	517	1284	5339	7230	NA^a^	193	900	1756	2978	134	169	441	5332	5727
**Neonatal outcomes (≤28 d after birth), *n* (%)**															
Admission to neonatal unit	112 (76)	458 (89)	792 (62)	399 (7.5)	591 (8.2)	NA	182 (94)	461 (51)	456 (26)	416 (14)	117 (87)	152 (90)	301 (68)	1411 (26)	638 (11)
Neonatal jaundice	40 (27)	184 (36)	581 (45)	436 (8.2)	573 (7.9)	NA	17 (8.8)	243 (27)	155 (8.8)	256 (8.5)	32 (24)	49 (29)	267 (61)	785 (15)	405 (7.1)
Congenital anemia	8 (5.4)	44 (8.5)	16 (1.2)	32 (0.6)	27 (0.4)	NA	6 (3.1)	≤5 (≤0.6)	10 (0.6)	11 (0.4)	15 (11)	11 (6.5)	19 (4.3)	21 (0.4)	9 (0.2)
Kernicterus	0 (0)	≤5 (≤1.0)	16 (1.2)	8 (0.1)	0 (0)	NA	0 (0)	7 (0.8)	≤5 (≤0.3)	≤5 (≤0.2)	≤5 (≤3.7)	≤5 (≤3.0)	≤5 (≤1.1)	≤5 (≤0.1)	≤5 (≤0.1)
Neonatal infections	8 (5.4)	55 (11)	42 (3.3)	161 (3.0)	241 (3.3)	NA	≤5 (≤2.6)	≤5 (≤0.6)	≤5 (≤0.3)	12 (0.4)	15 (11)	22 (13)	26 (5.9)	149 (2.8)	134 (2.3)
Cerebral palsy	0 (0)	0 (0)	0 (0)	0 (0)	0 (0)	NA	0 (0)	≤5 (≤0.6)	6 (0.3)	5 (0.2)	≤5 (≤3.7)	≤5 (≤3.0)	≤5 (≤1.1)	≤5 (≤0.1)	≤5 (≤0.1)
Visual impairments	0 (0)	0 (0)	0 (0)	0 (0)	≤5 (≤0.1)	NA	0 (0)	0 (0)	0 (0)	0 (0)	0 (0)	0 (0)	0 (0)	0 (0)	0 (0)
Hearing impairments	0 (0)	0 (0)	0 (0)	≤5 (≤0.1)	≤5 (≤0.1)	NA	0 (0)	0 (0)	0 (0)	0 (0)	≤5 (≤3.7)	≤5 (≤3.0)	≤5 (≤1.1)	≤5 (≤0.1)	≤5 (≤0.1)
Neonatal seizures	0 (0)	11 (2.1)	≤5 (≤0.4)	11 (0.2)	21 (0.3)	NA	0 (0)	0 (0)	0 (0)	≤5 (≤0.2)	≤5 (≤3.7)	≤5 (≤3.0)	≤5 (≤1.1)	≤5 (≤0.1)	18 (0.3)
Neonatal death	0 (0)	20 (3.9)	0 (0)	53 (1.0)	29 (0.4)	NA	≤5 (≤2.6)	0 (0)	23 (0.8)	14 (0.5)	0 (0)	0 (0)	0 (0)	0 (0)	0 (0)

Due to privacy regulations, values less than or equal to 5 are presented as “≤5.”

*IUT*, intrauterine transfusion.

a Data not available (NA).

**Table 5:** Long-term outcomes of liveborn children included in the study population, by treatment groups

	Sweden					Finland					Denmark				
	IUT	Transfusion	Phototherapy	Unknown	Non-HDFN	IUT	Transfusion	Phototherapy	Unknown	Non-HDFN	IUT	Transfusion	Phototherapy	Unknown	Non-HDFN
Total liveborn, *n*	149	517	1284	5339	7230	NA^a^	193	900	1756	2978	134	169	441	5332	5727
Median follow-up, y (IQR)	7.0 (3.3–11.9)	7.3 (3.3–11.2)	5.8 (2.4–9.9)	10.5 (6.3–15.8)	11.2 (6.4–15.8)	NA	17.1 (12.1–20.3)	10.5 (6.8–15.2)	12.4 (8.1–17.3)	14.3 (10.1–18.2)	7.4 (3.9–10.7)	10.8 (6.5–15.4)	6.1 (1.8–11.3)	10.9 (6.0–16.6)	11.8 (7.1–16.4)
**Long-term outcomes (>28d after birth), *n* (%)**															
Lack of normal physiological development	5 (3.4)	24 (4.6)	32 (2.5)	145 (2.7)	190 (2.6)	NA	6 (3.1)	30 (3.3)	56 (3.2)	54 (1.8)	12 (8.9)	12 (7.1)	17 (3.9)	258 (4.8)	235 (4.1)
ADHD	5 (3.4)	14 (2.7)	24 (1.9)	277 (5.2)	417 (5.8)	NA	11 (5.7)	36 (4.0)	88 (5.0)	153 (5.1)	≤5 (≤3.7)	≤5 (≤3.0)	≤5 (≤1.1)	≤5 (≤0.1)	19 (0.3)
Autism	≤5 (≤3.4)	10 (1.9)	21 (1.6)	112 (2.1)	150 (2.1)	NA	0 (0)	10 (1.1)	19 (1.1)	40 (1.3)	≤5 (≤3.7)	≤5 (≤3.0)	≤5 (≤1.1)	≤5 (≤0.1)	16 (0.3)
Infections	51 (34)	147 (28)	300 (23)	1398 (26)	2094 (29)	NA	30 (16)	149 (17)	317 (18)	557 (19)	35 (26)	42 (25)	82 (19)	1233 (23)	1254 (22)
Mental retardation	0 (0)	16 (3.1)	9 (0.7)	56 (1.0)	84 (1.2)	NA	≤5 (≤2.6)	8 (0.9)	15 (0.9)	18 (0.6)	≤5 (≤3.7)	≤5 (≤3.0)	≤5 (≤1.1)	≤5 (≤0.1)	10 (0.2)
Visual impairments	≤5 (≤3.4)	0 (0)	16 (1.2)	79 (1.5)	92 (1.3)	NA	≤5 (≤2.6)	≤5 (≤0.6)	6 (0.3)	7 (0.2)	≤5 (≤3.7)	≤5 (≤3.0)	≤5 (≤1.1)	≤5 (≤0.1)	12 (0.2)
Hearing impairments	≤5 (≤3.4)	19 (3.7)	17 (1.3)	87 (1.6)	100 (1.4)	NA	0 (0)	0 (0)	0 (0)	0 (0)	4 (3.0)	13 (7.7)	9 (2.0)	123 (2.3)	131 (2.3)
Disease of the nervous system	12 (8.1)	25 (4.8)	33 (2.6)	272 (5.1)	408 (5.6)	NA	17 (8.8)	49 (5.4)	102 (5.8)	208 (7.0)	7 (5.0)	13 (7.7)	8 (1.8)	261 (4.9)	279 (4.9)

Due to privacy regulations, values less than or equal to 5 are presented as “≤5.”

*ADHD*, attention-deficient hyperactive disorder; *IUT*, intrauterine transfusion.

a Data not available.

## Comment

### Principal findings

This study provides a comprehensive overview of maternal characteristics and outcomes of pregnancies at risk of HDFN, as well as the characteristics and outcomes of neonates born to these pregnancies in Sweden, Finland, and Denmark. Multiparity and caesarean deliveries were more common in HDFN pregnancies compared to non-HDFN pregnancies. Notable variations in perinatal treatment patterns for HDFN were observed across countries. HDFN neonates were more likely admitted to neonatal units. In Sweden and Denmark, HDFN neonates who received IUT or transfusions had a higher incidence of infection compared to non-HDFN neonates. In the long-term, a larger proportion of children who underwent IUT or neonatal transfusions were diagnosed with impaired physiological growth and nervous system disorders than those born to non-HDFN pregnancies.

### Results in the context of what is known

To the best of our knowledge, this population-based cohort study is the first to comprehensively describe the clinical characteristics and outcomes of HDFN in multiple countries that have similar national tax-funded healthcare systems.

Compelling evidence^8^, ^9^, ^10^, ^11^, ^12^ has shown multiparity to be a strong risk factor for RBC alloimmunization, which is corroborated by our findings in all countries. Low stillbirth rates reported in this study are consistent with published results from Nordic countries.^11^^,^^13^^,^^14^ The higher occurrence of caesarean delivery, preterm births, and low birth weight/length observed in HDFN pregnancies, particularly those treated with IUT and transfusion, are also in line with the literature,^15^^,^^16^ which may be a consequence of the recommended antenatal management for severe HDFN cases.^14^^,^^17^

The significant clinical burden of HDFN on pregnancies was reflected by the substantial number of neonates that required IUTs, neonatal transfusions, and/or phototherapy. However, we found a large discrepancy in the proportion of HDFN neonates receiving the same treatments in different countries. Given that previous studies have also reported large variances in the rate of transfusions and, to a lesser extent, phototherapy in alloimmunized pregnancies,^6^ our findings suggest a lack of consensus on treatment threshold or differences in coding practice across the three countries. Alternative treatment options are available, including prenatal IVIg,^18^ which has been mentioned in management guidelines for alloimmunized pregnancies in all three countries. But due to the limited evidence on its effects on HDFN, it is currently not recommended as a routine therapy, but rather on a case-by-case basis for delaying the need of IUT in severe cases after individual risk assessment. However, this data was not systematically reported in nationwide registers across the three countries and hence could not be included in this study. Therefore, the possibility of perinatal exposure to IVIg, especially in the unknown treatment group, cannot be ruled out.

To date, few studies have explored the long-term clinical outcomes of HDFN beyond the neonatal period.^19^ In the present study, longitudinal register data was used to follow neonates into their childhood over an extended period (median follow-up of up to 17 years). In agreement with previous studies focusing on neurological and behavioral sequelae in severe cases requiring IUT treatment,^20^^,^^21^ long-term outcomes in IUT-treated children in Sweden and Denmark were largely similar to non-HDFN children. It should be noted that our results focused only on outcomes known to be associated with HDFN, or related conditions, namely hyperbilirubinemia and kernicterus.^5^^,^^21^^,^^22^ Further studies are required to characterize the full spectrum of long-term sequelae in these children.

### Clinical implications

Our findings revealed variations in the study populations across the Nordic countries. Although Finland and Denmark have populations about half the size of Sweden, the number of at-risk pregnancies identified in Denmark was unexpectedly double that of Finland and nearly comparable to Sweden. This larger cohort in Denmark seemed to result from a higher rate of prenatal diagnoses of maternal care for alloimmunization or fetal hydrops (Table 1), which was not reflected in postnatal HDFN diagnoses. This may be due to more sensitive prenatal screening and intensive monitoring in Denmark, consistent with the lower postnatal treatment rates observed in Danish HDFN neonates compared to Sweden and Finland. Differences in the timing of implementing national screening programs for antenatal anti-D prophylaxis may also explain these variations. Denmark introduced a screening program in 2010^23^ followed by Finland in 2014.^24^ Sweden evaluated the impact of a similar program between 2009 and 2011,^25^ which was implemented in routine practice in 2012 in the Stockholm area, then sequentially introduced in different health regions throughout the country. These screening programs could also be a possible reason for a drop in the number of pregnancies captured in later calendar years across the countries in the present study due to better preventive care. More investigations are required to understand to what extent this occurs in each country. Besides, despite the similarities in clinical guidelines across these countries in terms of preventive measures, noninvasive monitoring, and planned delivery in affected pregnancies, they differ in various aspects, such as the exact timing for alloantibody screening, risk classification by antibody type and titer, as well as antenatal follow-up schedules, which could be a reason for the variations observed, but this remains to be confirmed. Another potential factor contributing to the discrepancy may be the inconsistency in the registration criteria of these diagnosis codes across countries, which could entail a range of hospital activities from visits for routine checkup to sampling for lab measurements and administration of prophylaxis. In addition, the reason for the particularly lower neonatal mortality in Denmark was not completely understood. Variations in healthcare infrastructure and routine screening and clinical practice^26^ could be a factor, or it could be a chance finding. Further comparative analyses evaluating the routine practice in each country will provide a better understanding of treatment decisions.

Neonates in the transfusion group across all countries exhibited poor clinical characteristics at birth and had higher rates of admission to neonatal units, even when compared to the more severe cases treated with IUT. However, data on the indications for neonatal unit admission was not available. Nevertheless, this suggests a significant unmet need in managing less severe forms of HDFN. Possible reasons include inadequate antenatal monitoring, underrecognition of at-risk pregnancies, or a lack of urgency to refer to specialist centers. Future studies focusing specifically on the clinical characteristics and outcomes of these transfusion-treated neonates will be valuable for understanding their needs and improving care.

### Research implications

This study underscores the necessity for more comprehensive and standardized reporting of perinatal treatments, including IUT, IVIg, and anti-D prophylaxis, to enhance the comparability and reliability of research findings across different healthcare settings. The absence of granular data on treatment-related adverse events limits the assessment of safety profiles and therapeutic efficacy, highlighting an area for improvement in future studies. Additionally, more studies are needed to explore the temporal changes in treatment patterns in relation to fetal and neonatal outcomes, which is critical for understanding the evolution and long-term impact of contemporary clinical practices. Furthermore, detailed characterization of IUT and transfusion episodes are warranted to evaluate cost-effectiveness and optimize clinical management strategies for more severe cases. Addressing these research gaps will provide a more comprehensive understanding of the perinatal treatment landscape and inform evidence-based guidelines for managing pregnancies at risk of HDFN.

### Strengths and limitations

This study’s strengths include its large sample size and multi-country design, which provides a comprehensive and generalizable account on the perinatal management and outcomes of the disease. Secondly, the utilization of nationwide population-based registers and robust mother-child linkage ensures thorough and accurate data capture, minimizes the selection and recall biases, and enhances the validity of the study findings. Thirdly, the complete follow-up of pregnancies from prenatal to neonatal period and beyond offers valuable insights into the continuum of care and long-term influences of HDFN.

Despite its strengths, the study has several limitations. First, the reliance on proxy measures, such as the use of ICD-10 code O36.2, which includes both immune and nonimmune fetal hydrops, to define at-risk pregnancies may have introduced misclassification, potentially affecting the accuracy and interpretability of our findings. Besides, the use of proxies limited the possibility to determine the exact gestational age at diagnosis. Additionally, the absence of IUT data from Finnish health registers limits our ability to fully compare this treatment modality across countries and likely leads to an underestimation of severe HDFN cases in Finland.^15^ There is also the possibility of underreporting milder HDFN cases and related treatments in the registers, which may result in an underestimation of the true prevalence and outcomes. Moreover, beyond the treatments examined in this study, the lack of systematic reporting of other potential perinatal therapeutic modalities such as IVIg and anti-D prophylaxis limited the ability of this study to describe a complete patient journey. Furthermore, we lacked access to detailed data on treatment complications and adverse events, preventing us from assessing the safety profiles and therapeutic effectiveness of different treatment approaches. Finally, our dataset did not have sufficient information to describe previous alloimmunization events or passive cases of HDFN, or number of IUT required per patients, to depict a more complete clinical picture of the study cohort. The study period of the Danish cohort was shifted 3 years earlier compared to the Finnish and Swedish cohorts due to data availability. Given the same total timespan of 20 years in all countries, this likely had limited impact on the findings of this observational study.

It is worth mentioning that while parity and gestational age differed between HDFN neonates and their unaffected siblings (non-HDFN neonates), these factors could not explain most of the associations because of the magnitude and direction of the outcomes of interest. For some outcomes, gestational age at birth is a potential intermediate variable that could explain part of our findings. However, we performed no adjustment for this potential intermediate variable because of the risk of collider bias, and because the clinical aim of the study was to describe the possible effects of the current clinical practice. In this study, the purpose of including affected siblings in the analyses was to contextualize the medical burden observed in HDFN neonates, in particular the phototherapy and unknown treatment groups, which may be interpreted as the milder cases of HDFN.

## Conclusions

This study provides a detailed overview of pregnancies at risk of HDFN using comprehensive population-based register data from Sweden, Finland, and Denmark. The findings show notable differences in maternal characteristics and delivery method between HDFN and non-HDFN (unaffected siblings) pregnancies. Neonates with HDFN, particularly those requiring IUT or neonatal transfusions, showed poorer outcomes at birth compared to those without HDFN. Further research is warranted to evaluate current clinical practice and to improve the outcomes of at-risk pregnancies.

## CRediT authorship contribution statement

**Kelvin H.M. Kwok:** Writing – review & editing, Writing – original draft, Project administration, Methodology, Formal analysis, Data curation, Conceptualization. **Mika Gissler:** Writing – review & editing, Supervision, Methodology, Formal analysis, Data curation. **Mette Ø. Thunbo:** Writing – review & editing, Methodology, Formal analysis, Data curation. **Elizabeth C. Hsia:** Writing – review & editing, Methodology, Conceptualization. **May Lee Tjoa:** Writing – review & editing, Methodology. **Shengxin Liu:** Writing – review & editing, Writing – original draft, Methodology, Formal analysis, Data curation. **Malin Almgren:** Writing – review & editing, Project administration, Methodology, Conceptualization. **Vedran Stefanovic:** Writing – review & editing, Methodology. **Lars H. Pedersen:** Writing – review & editing, Supervision, Methodology, Formal analysis, Data curation. **Agneta Wikman:** Writing – review & editing, Supervision, Resources, Methodology.

## Appendix Supplementary materials

Image, application 1

## Footnotes

Supplementary material associated with this article can be found in the online version at doi:10.1016/j.xagr.2025.100544.
