# Supplementary material for: Clinical characteristics and outcomes of pregnancies at-risk of hemolytic disease of the fetus and newborn in Sweden, Finland, and Denmark: a population-based register study
Source: AJOG Glob Rep. 2025 Jul 8;5(3):100544. doi: 10.1016/j.xagr.2025.100544 (PMC12337666; doi:10.1016/j.xagr.2025.100544)
Supplement: Supplementary file 1 [file mmc1.docx]

**Clinical Characteristics and Outcomes of Pregnancies At-risk of HDFN in Sweden, Finland, and Denmark: A Population-based Register Study**

**SUPPLEMENTAL INFORMATION**

**Data Sources**

***Sweden***

*Medical Birth Register* contains data on births in Sweden since 1973 with a nationwide coverage, including mothers’ demographic characteristics, disease history, parity, pregnancy and delivery information, and neonatal details. All diagnoses are coded using ICD-10 codes.

*Patient Register* contains information on inpatient (since 1987) and outpatient specialist (since 2001) care, including patient admission and discharge dates, and the diagnosis codes (International Classification of Diseases 10^th^ version, ICD-10) and procedure codes associated with each care visit. Medications dispensed within the hospital are coded using Anatomical Therapeutic Chemical (ATC) codes.

*Swedish Neonatal Quality Register* *(SNQ)* is a quality register that contains data on neonates admitted to neonatal units within 27 days of birth, including disease diagnosis (ICD-10 code) and neonatal treatment. Data collection started in 2001 and reached a national coverage in 2012.

*Cause of Death Register* records death information for all individuals resided in Sweden, including the date of death.

*GravImm* contains data on erythrocyte-alloimmunized pregnancies since 1990 (retrospectively data collection before 2008, and prospectively thereafter), including prenatal treatment such as intrauterine transfusion (IUT, with nationwide coverage), and maternal antibody type and titers.

***Finland***

*Medical Birth Register* was established in 1987 and collects data related to maternity care, obstetrical services, and neonatal care, including pregnancy outcomes, perinatal treatment, and maternal diagnoses (ICD-10).

*Care Register for Health Care (HILMO)*, established in 1967, contains information on all inpatient hospital admissions and discharges with the associated diagnoses (ICD-10 code) and medication (ATC code).

*Social Insurance Institution (KELA)* handles health insurance to provide coverage for sickness-related expenses since 1990s and contains data on reimbursement decisions and the associated disease diagnoses (ICD-10 code), prescription of reimbursed medications (ATC code), and information on medical expenses.

*Cause of Death Register* was established since 1936 and collects data on the causes of death together with age, gender and other demographic information for all persons resided in Finland.

***Denmark***

*Medical Birth Register* contains data related to maternity and neonatal care (since 1973), including pregnancy outcomes, perinatal treatment, and maternal diagnoses (ICD-10 code).

*National Patient Register* contains information on all inpatient hospital admissions and discharges (since 1977), together with the associated disease diagnoses (ICD-10 code).

*National Health Services Prescription Database* contains detailed information on reimbursed prescriptions dispensed at community pharmacies in Denmark since 1995, including date and type of medication (ATC code), package size and the number of package dispensed.

*Cause of Death Register* collects data on the causes of death together with age, gender and other demographic information for all individuals reside in Denmark. The register began operation since 1875, and records were computerized since 1970.

**Supplemental Table 1. Inclusion criteria of the study population.**

|  | **Description** | **ICD-10** |
| --- | --- | --- |
| **Pregnancies monitored or treated for potential alloimmunization** | Maternal care for rhesus isoimmunization | O36.0 |
|  | Maternal care for other isoimmunization | O36.1 |
|  | Maternal care for hydrops fetalis | O36.2 |
| **Postnatal diagnosis of HDFN-related conditions** | Rh isoimmunization in fetus or newborn | P55.0 |
|  | Other hemolytic diseases of fetus or newborn | P55.8 |
|  | Hemolytic disease of fetus or newborn, unspecified | P55.9 |
|  | Hydrops fetalis due to isoimmunization | P56.0 |
|  | Kernicterus due to isoimmunization | P57.0 |

**Supplemental Table 2. Categorization of the included pregnancies.**

| **Treatment group** | | **Sweden** | **Finland** | **Denmark** |
| --- | --- | --- | --- | --- |
| IUT | | Recorded in GravImm | NSCP: MAA34 | SKS: KMAA30A |
| Neonatal transfusion (<28d after birth) | Blood transfusion | KVÅ: DP029  Recorded in SNQ | NSCP: WW500  NSCP: TPH61 | SKS: BOQA0  SKS: BOQA5 |
|  | Exchange transfusion | KVÅ: DR050  Recorded in SNQ |  |  |
| Neonatal phototherapy  (<28d after birth) | | KVÅ: DQ015  Recorded in SNQ | NSCP: WXQ24 | SKS: BNGC0 |

IUT, intrauterine transfusion; KVÅ, classification of care measures; SNQ, Swedish Neonatal Quality Register; NSCP, NOMESCO Classification of Surgical Procedures; SKS, Health Service Classification System.

**Supplemental Table 3. Diagnostic criteria or/and ICD-10 codes for the examined outcomes.**

| **Outcome** | **Sweden** | **Finland** | **Denmark** |
| --- | --- | --- | --- |
| **Maternal complications** | | | |
| Gestational hypertension | O13 | O13 | O13 |
| Gestational diabetes | O24.4 | O24.4 | O24.4 |
| Proteinuria during pregnancy | O12.1, O12.2 | O12.1-O12.2 | O12.1, O12.2 |
| Kidney disease associated with pregnancy | O08.4, O26.8, O90.4 | O08.4, O26.8 | O08.4, O26.8C, O904 |
| Preeclampsia | O14 | O14 | O14 |
| Eclampsia | O15 | O15 | O15 |
| Placental abruption | O45 | O45 | O45 |
| Placenta previa | O44 | O44 | O44 |
| Preterm premature rupture of membranes | O42 | O42 | O42 |
| Obstetric embolism | O88 | O88 | O88 |
| Intrauterine growth restriction | O36.5 | O36.5 | DO36.5, DO368, DO368A, DO368E, DO368E1, DO368E2, DO368F, DO368G |
| **Neonatal and long-term diagnosis** | | | |
| Small for gestational age (SGA) | P05, P07 | P05, P07 | P05, P07 |
| IUGR-associated SGA | O36.5 + P05/P07 | O36.5 + P05/P07 | O36.5 + P05/P07 |
| Neonatal jaundice | P58, P59 | P58-P59 | P58-P59 |
| Kernicterus | P57 | P57 | P57 |
| Congenital anemia | P61.3, P61.4 | P61.3-P61.4 | P61.3-P61.4 |
| Infections | P36, P39.9, A00-B99 | P36, P39.9, A00-B99 | P36, P39.9, A00-B99 |
| Cerebral palsy | G80 | G80 | G80 |
| Visual impairment | H54 | H54 | H54 |
| Hearing impairment | H90, H91 | H90-H91 | H90-H91 |
| Neonatal seizure | P90 | P90 | P90 |
| Lack of expected normal physiological development | R62 | R62 | R62 |
| Attention deficit hyperactivity disorder (ADHD) | F90 | F90 | F90 |
| Autism | F84 | F84 | F84 |
| Infections | J12-J18, A00-B99 | J12-J18, A00-B99 | J12-J18, A00-B99 |
| Mental retardation | F70-F79 | F70-F79 | F70-F79 |
| Disease of the nervous system | G00-G99 | G00-G99 | G00-G99 |

Key maternal characteristics included maternal age at delivery, parity, and maternal BMI, maternal complications (gestational hypertension, gestational diabetes, proteinuria during pregnancy, kidney disease associated with pregnancy, preeclampsia, eclampsia, placental abruption, placenta previa, preterm premature rupture of membranes, and obstetric embolism), prenatal diagnosis (intrauterine growth restriction [IUGR]), delivery methods (vaginal and caesarean sections, with caesareans further divided into elective and emergency), and pregnancy outcomes (live births and stillbirths).

Characteristics at birth covered the infant's sex, gestational age, birth weight, birth length, Apgar scores at 1 and 5 minutes, instance of small for gestational age (SGA) and IUGR-associated SGA.

Neonatal outcomes included neonatal jaundice, kernicterus, anemia, congenital anemia from fetal blood loss, severe and perinatal infections, cerebral palsy, neonatal unit admissions (with length of stay recorded in Sweden and Denmark), visual and hearing impairments, and seizures (such as epilepsy, status epilepticus, and convulsions in newborns). Long-term health outcomes were also scrutinized, focusing on developmental delay, attention-deficit/hyperactivity disorder (ADHD), autism, mental retardation, recurrent severe infections, and persistent visual and hearing impairments.

**Supplemental Table 4. Pre/postnatal diagnoses of liveborn neonates included in the study population, by treatment groups.**

|  | **HDFN** | | | |
| --- | --- | --- | --- | --- |
| **Diagnosis, n (%)** | **IUT** | **Transfusion** | **Phototherapy** | **Unknown** |
| **Sweden** | | | | |
| Total HDFN liveborn children | 149 | 517 | 1284 | 5339 |
| Prenatal care only^a^ | 7 (5) | 79 (15) | 318 (25) | 3856 (72) |
| Postnatal care only^b^ | 0 (0) | 112 (22) | 587 (46) | 754 (14) |
| Pre- and postnatal care | 142 (95) | 326 (63) | 379 (30) | 729 (14) |
| **Finland** | | | | |
| Total HDFN liveborn children | NA^c^ | 193 | 900 | 1756 |
| Prenatal care only | NA | 16 (8) | 209 (23) | 1231 (70) |
| Postnatal care only | NA | 62 (32) | 344 (38) | 294 (17) |
| Pre- and postnatal care | NA | 115 (60) | 347 (39) | 231 (13) |
| **Denmark** | | | | |
| Total HDFN liveborn children | 134 | 169 | 441 | 5332 |
| Prenatal care only | . | . | 128 (29) | 4040 (76) |
| Postnatal care only | . | . | 126 (29) | 602 (11) |
| Pre- and postnatal care | 106 (79) | 115 (68) | 187 (42) | 690 (13) |

^a ‘^Prenatal care’ includes prenatal diagnoses of maternal care for alloimmunization or hydrops (ICD-10: O36.0-O36.2).

^b^ ‘Postnatal care’ includes postnatal diagnoses of HDFN-related conditions (ICD-10: P55.0, P55.8-9, P56.0, P57.0).

^c^ Data is not available (NA)

HDFN, hemolytic disease of fetus and newborn; IUT, intrauterine transfusion.

Due to data privacy regulations, reported values ≤5 or those that reveal true ≤5 values are shown as ‘.’.

**Supplemental Figure 1.** **Inclusion criteria for the study population.**

Singleton pregnancies (despite pregnancy outcome) are stratified into “HDFN” and “non-HDFN”.

For pregnancies, a HDFN case is defined as either or both of the following:

- Liveborn children with postnatal diagnosis of HDFN-related conditions (#3, #4, and #6)
- Mothers, who were monitored or treated for alloimmunization during the pregnancy despite pregnant outcome (miscarriage, stillbirth, or liveborn) (#1 and #3)

Liveborn children are stratified into two groups: “HDFN” and “non-HDFN”.

A HDFN case is defined as a child:

- With a postnatal diagnosis of HDFN-related conditions, and/or
- Whose mother was monitored or treated for alloimmunization during this particular pregnancy

Hence, in Supplementary Figure 1, Pregnancy-Child pairs #1, 3, 4, and 6 are classified as 'HDFN', whereas #2 and 5 are classified as 'No HDFN'.
